# Relation between resting amygdala activity and cardiovascular events in patients with cardiac sarcoidosis

**DOI:** 10.1007/s00259-025-07266-3

**Published:** 2025-04-14

**Authors:** Sou Otsuki, Masahiro Hatakeyama, Atsushi Michael Kimura, Kosei Nakamura, Mikhail Ratanov, Rie Akagawa, Hironori Furuse, Naomasa Suzuki, Yasuhiro Ikami, Yuki Hasegawa, Masaomi Chinushi, Hitoshi Shimada, Takayuki Inomata

**Affiliations:** 1 Department of Cardiovascular Biology and Medicine, Niigata University Graduate School of Medical and Dental Sciences, 1-757 Asahimachidori, Chuo-ku, Niigata, 951-8510 Japan; 2 Department of Functional Neurology & Neurosurgery, Center for Integrated Human Brain Science, Brain Research Institute, Niigata University, 1-757 Asahimachidori, Chuo-ku, Niigata, 951-8510 Japan; 3 Niigata University School of Medicine, Niigata, Japan

**Keywords:** Amygdala, Stress-related neural activity, Cardiac sarcoidosis, VT/VF, 18F-FDG-positron emission tomography

## Abstract

**Purpose:**

Previous studies indicate amygdala activity (AmygA) measured by FDG-positron emission tomography (PET) predicts the risk of subsequent cardiovascular events. However, AmygA measurement use for predicting the prognosis of patients with cardiac sarcoidosis (CS) is unknown. We aimed to investigate the association between AmygA and cardiovascular events in patients with CS.

**Methods:**

Consecutive 40 patients with CS who underwent FDG-PET were identified retrospectively. Cardiovascular events included ventricular tachyarrhythmias and heart failure hospitalizations. We examined the association between AmygA and cardiovascular events and investigated the timing of FDG-PET influence on AmygA measurements.

**Results:**

During a follow-up of 4.5 (2.5–7.7) years, 14 (35%) patients experienced cardiovascular events. Left-AmygA was a stronger cardiovascular event predictor than the Right-AmygA, and incidences were significantly higher in the high left-AmygA group than low group (log-rank *P* = 0.018). Multivariable analysis revealed left-AmygA augmentation (hazard ratio [HR]: 1.76 per 0.1 increase, 95% confidence interval [CI]: 1.12–2.87, *P* = 0.016) was the only independent predictor of cardiovascular event. Among the 40 patients, 32 underwent multiple PET-scans. No significant difference was found between the AmygA value on the first PET-scan and the mean on multiple PET scans, and the correlation coefficient was 0.93 (*P* < 0.001). Multivariate analyses revealed the mean left-AmygA value was the only independent predictor of cardiovascular event (*P* = 0.011).

**Conclusions:**

High left-AmygA was associated with the higher rates of cardiovascular events in patients with CS. AmygA would be a prognostic biomarker regardless of the PET-scan timing.

**Supplementary Information:**

The online version contains supplementary material available at 10.1007/s00259-025-07266-3.

## Introduction

Sarcoidosis is a granulomatous disease of unknown cause, and in rare cases, it can damage the cardiac muscle. Cardiac sarcoidosis (CS) is generally treated with steroids. However, even with drug treatment, fatal cardiac events such as ventricular tachyarrhythmia can occur, making treatment with implantable cardioverter defibrillator (ICD) unavoidable. Five-year survival rates in recent cohorts of cardiac sarcoidosis are consistently 90% or higher, and Nordenswan et al. reported that 72% of deaths are owing to cardiac events such as heart failure or sudden cardiac death [1]. To improve the prognosis of CS, predicting the risk of developing cardiac events at an early stage, and to make appropriate medication adjustments and perform early ICD implantation is necessary. However, no reports are available on established biomarkers that predict the prognosis of cardiac events of CS.

A previous study indicated that altered autonomic nervous system function characterized by autonomic imbalance (i.e., relatively high sympathetic activity and relatively low parasympathetic activity) is one of the mechanisms underlying the increased risk for adverse somatic health outcomes, such as cardiovascular disease, hypertension, diabetes, and stroke as well as all-cause mortality [2, 3]. A close association was observed between ventricular arrhythmias and sympathetic activity, as evidenced by the diurnal variation in ventricular arrhythmia occurrence and sedation efficacy for electrical storms [4, 5, 6].

The amygdala’s efferent projections to the brainstem participate in the sympathetic responses to stress [7]. Amygdala activity (AmygA) measured with 2-[^18^F]fluoro-2-deoxy-D-glucose (FDG)-positron emission tomography (PET) is a known alternative for stress-related neural activity. Recent studies have emphasized the role of the left amygdala—a region involved in processing stress responses—in predicting subsequent cardiovascular events [8, 9, 10, 11]. However, no reports investigated the association between AmygA and cardiovascular events, mainly ventricular tachyarrhythmias, in specific nonischemic cardiomyopathy.

We hypothesized that in patients with CS, an increase in AmygA is associated with the increased risk of cardiovascular events, and that evaluating AmygA using FDG-PET could be a prognostic biomarker in cardiac sarcoidosis. Herein, we retrospectively investigated the association between AmygA measured by FDG-PET and cardiovascular events in patients with CS. Furthermore, we examined the clinical features related with high-AmygA among patients.

## Methods

### Study design and population

This was a single-center retrospective study. We included 40 consecutive patients diagnosed with CS and those who underwent FDG-PET during steroid therapy from January 2009 to May 2023. We confirmed that all patients met either the histological or clinical diagnosis criteria of CS following the latest guidelines from the Japanese Circulation Society (JCS) [12]. Isolated CS was diagnosed if they met the diagnostic criteria in the latest JCS guidelines even in patients without histological evidence of CS in organs other than the heart [12].

As the main objective, we investigated the hypothesis that high AmygA levels in patients with CS could be a poor prognosis marker. The right and left-AmygA levels and the mean of the bilateral AmygA levels were investigated for their association with cardiovascular events. Cardiovascular events included ventricular tachyarrhythmias (VT/VF) and worsening heart failure that require hospitalization. VT/VF were confirmed by appropriate ICD therapy records or sustained VT/VF documented by ECG or a cardiovascular implantable electronic device (CIED).

In patients who underwent multiple FDG-PET scans during the course of treatment, we also examined whether AmygA evaluated by FDG-PET showed any fluctuation during the disease course.

The Institutional Review Board of Niigata University Medical & Dental Hospital, Niigata, Japan approved this study (2024-0064), conducted under the Declaration of Helsinki.

### FDG-PET imaging protocol and measurement of the amygdala activity

FDG was intravenously administered at a 2–5 MBq/kg dosage after an overnight fast. The patients rested after tracer injection, and PET imaging was conducted after approximately 60 min with an integrated scanner (Biograph mCT 20, Siemens Healthineers, Erlangen, Germany). A nongated, noncontrast-enhanced CT scan (120 kV, ∼ 80 mAs) was conducted for attenuation correction. Whole-body scans were cropped for the skull, and a quantitative assessment of resting amygdalar activity was performed. Serial regions of interest (ROI) were placed in the left and right amygdala, and pons. ROIs of the amygdala were delineated based on the AAL-VOI atlas preinstalled in the PMOD (version 4.3, PMOD Technologies Ltd, Fällanden, Switzerland) per previous report [13]. Further, the mean standardized uptake value ratio of FDG was calculated as the ratio of each amygdala to the pons [14, 15].

### Clinical data and device programming and management

Patients’ characteristics at the time of diagnosis of CS were used as baseline data. Seven (18%) patients had pacemakers implanted for atrioventricular block, 14 (35%) had ICD/CRT-D implanted for primary prevention, and 7 (18%) for secondary prevention. In the 21 (53%) patients with ICD/CRT-D, VT/VF therapies by ICD/CRT-D were programmed according to the opinion of the attending physicians who considered the patient’s clinical status. Ultimately, the lower limit of the VT/VF detection zone was set as ≥ 150 bpm in all patients, except for one patient (in whom a slow VT had been documented). In seven patients with pacemakers, the monitoring zone for recording sustained VT/VF was programmed to 150–200 bpm.

### Statistical methods

Intergroup differences in clinical characteristics and course were identified using the unpaired *t*-test for continuous variables and χ^2^ test for categorical variables. Quantitative data were expressed as mean ± standard deviation (SD), median and interquartile range, and ranges based on data distribution and compared with Student’s *t*-test for normally distributed data and Mann–Whitney U test for nonparametric data. The incidence rates were estimated with Kaplan–Meier analysis using the date of CS diagnosis as the starting point and compared with the log-rank test for all time-to-event analyses. Cox regression analysis was conducted to assess the association between cardiovascular events and patient characteristics, including AmygA, and odds ratios were reported along with their 95% confidence intervals (CIs) (multivariate analysis included variables with *p*-values of < 0.05 in the univariate model).

The correlation coefficient between the AmygA values in the first PET and the mean AmygA values in multiple PET was calculated to investigate the variation of AmygA values in multiple PET scans. Corresponding *t*-tests were conducted to investigate the presence of a significant difference between the initial value and the mean value multiple times.

A *p*-value of < 0.05 indicated a statistically significant difference between the groups. Categorical data are presented as frequencies and percentages. Continuous data are described as mean ± SD.

## Results

### Clinical characteristics

Table 1 summarizes the baseline data of the patients. The median age was 63.5 (59–69) years, and 29 (73%) were women. A CIED was implanted in 28 patients, including 21 ICD/CRT-D (secondary prevention in 6) at baseline. All the patients reported no history of neurological or psychiatric disease.

**Table 1:** Comparison of patient characteristics between cardiovascular event (+) and (-) groups

	Cardiac event (+) *n* = 14	Cardiac event (-) *n* = 26	*P* value
Age, years	63.0 (55.3–69.5)	64.5 (59.8–69.0)	0.79
Male, *N* (%)	4 (29%)	7 (27%)	1.0
Height, cm	159.9 ± 8.3	158.4 ± 8.9	0.60
Weight, kg	55.1 ± 5.7	60.3 ± 10.0	0.051
Body mass index, kg/m2	21.7 (20.3–22.7)	22.2 (20.9–26.5)	0.14
**Systolic blood pressure**, **mmHg**	**97 (90–108)**	**116 (96–124)**	**0.028**
Heart rate, bpm	68.9 ± 9.6	64.8 ± 10.7	0.23
Therapy			
Maintenance dose of corticosteroids, mg/d	5.0 (5.0-6.3)	5.0 (5.0-5.6)	0.81
B-blocker, *N* (%)	7 (50%)	13 (50%)	1.0
ACE inhibitor/ARB, *N* (%)	6 (43%)	13 (50%)	0.75
Antiarrhythmic drugs class III, *N* (%)	2 (14%)	1 (4%)	0.28
PM, *N* (%)	1 (7%)	6 (23%)	0.39
ICD, *N* (%)	**8 (57%)**	**3 (12%)**	**0.007**
CRT-D, *N* (%)	4 (29%)	6 (23%)	0.72
Comorbidity			
Hypertension, *N* (%)	5 (36%)	11 (42%)	0.75
Chronic Kidney Disease, *N* (%)	4 (29%)	6 (23%)	0.72
Diabetes mellitus, *N* (%)	1 (7%)	6 (23%)	0.39
Dyslipidemia, *N* (%)	3 (21%)	11 (42%)	0.30
Atrial fibrillation, *N* (%)	2 (14%)	4 (15%)	1.0
NSVT, *N* (%)	3 (21%)	3 (12%)	0.65
Complete AVB, *N* (%)	6 (43%)	13 (50%)	0.75
History of VT/Vf, *N* (%)	**6 (43%)**	**2 (8%)**	**0.014**
Heart failure hospitalization, *N* (%)	2 (14%)	4 (15%)	1.0
CS diagnosis			0.65
Histological, *N* (%)	3 (21%)	3 (12%)	
Clinical, *N* (%)	11 (79%)	23 (88%)	
Abnormal uptake of ^18^F-FDG PET in the heart, *N* (%)	2 (14%)	2 (8%)	0.60
LV ejection fraction, (%)	42.8 ± 12.9	47.6 ± 16.2	0.36
ECG findings at baseline			
Corrected QT interval, ms	483.8 ± 31.0	461.1 ± 42.3	0.094
QRS duration, ms	145.4 ± 25.9	139.5 ± 30.1	0.55

Mean ± S.D

ACE inhibitor/ARB, Angiotensin Coverting Enzyme Inhibitor/angiotensin receptor blocker; AmygA, amygdala activity; AVB, atrioventricular block; CRT, Cardiac resynchronized therapy; CS, cardiac sarcoidosis; ECG, electrocardiography; ICD, Implantable Cardioverter Defibrillator; LV, left ventricular; NSVT, non-sustained ventricular tachycardia; PM, pacemaker; VT, ventricular tachycardia; Vf, ventricular fibrillation

During a median follow-up duration of 4.5 (2.5–7.7) years, nine patients developed VT/VF, two were hospitalized for heart failure, and three patients experienced VT/VF and heart failure hospitalization. Patients were categorized into two groups based on the presence or absence of cardiovascular events to compare their characteristics. In the patients with cardiovascular events, a history of VT/VF and of ICD implantation were more common and systolic blood pressure was lower.

### Association of amygA with cardiovascular events and variation of amygA

The median values of AmygA for the right, left, and mean of both sides in all patients were 1.11 (1.06–1.19), 1.10 (1.02–1.22), and 1.10 (1.06–1.18), respectively. Table 2 depicts a comparison between the cardiovascular events (+) and (−) patients in terms of AmygA data, and Supplemental Fig. 1 presents the distribution of AmygA values in all patients. AmygA values on the first PET were significantly higher in patients with cardiovascular events (+) than in those with cardiovascular events (−), whether on the right side, left side, or bilateral averages (*P* = 0.045, 0.013, and 0.027, respectively).

**Table 2:** Comparison of amygdala activity data between cardiovascular event (+) and (-) groups

	Cardiac event (+)	Cardiac event (-)	*r* value	*P* value
Amygdala activity value on initial PET-CT (*N* = 40)				
Right	**1.171 (1.086–1.308)**	**1.093 (1.042–1.148)**	**0.32**	**0.045**
Left	**1.179 (1.074–1.303)**	**1.081 (1.006–1.109)**	**0.38**	**0.017**
Mean of both	**1.152 (1.083–1.308)**	**1.086 (1.031–1.141)**	**0.35**	**0.027**
Mean amygdala activity value on multiple PET-CT (*N* = 32)				
Right	1.177 (1.128–1.212)	1.115 (1.068–1.154)	0.30	0.056
Left	**1.183 (1.057–1.254)**	**1.045 (1.021–1.137)**	**0.32**	**0.042**
Mean of both	1.166 (1.085–1.234)	1.077 (1.047–1.138)	0.31	0.051

Abbreviations are shown in Table 1. PET; positron emission tomography

Among the 40 patients, 32 underwent multiple PET scans (median: 3 [2, 3, 4]). The time from the first PET to the last PET was 2.0 [0.7–3.4] years. No significant difference was found between the AmygA value on the first PET scan and the mean AmygA value on multiple PET scans (Supplemental Fig. 2). The correlation coefficient between the AmygA value on the first CT scan and the mean AmygA value on multiple PET scans was 0.93 (*P* < 0.001). The mean AmygA values on multiple PET scans were higher in patients with cardiovascular events than in those without cardiovascular events on the left side and tended to be higher on the right side and bilateral averages (Table 2). The AmygA value on the first PET scan was strongly correlated with the mean AmygA value on multiple PET scans; thus, the following analyses were conducted using the AmygA value on the first PET scan.

### High left-AmygA is related to cardiovascular events

The largest difference in AmygA was observed on the left side comparing the patients with and without cardiovascular events. Therefore, we investigated the association between left-AmygA on first PET and cardiovascular events.

**Fig. 1:**
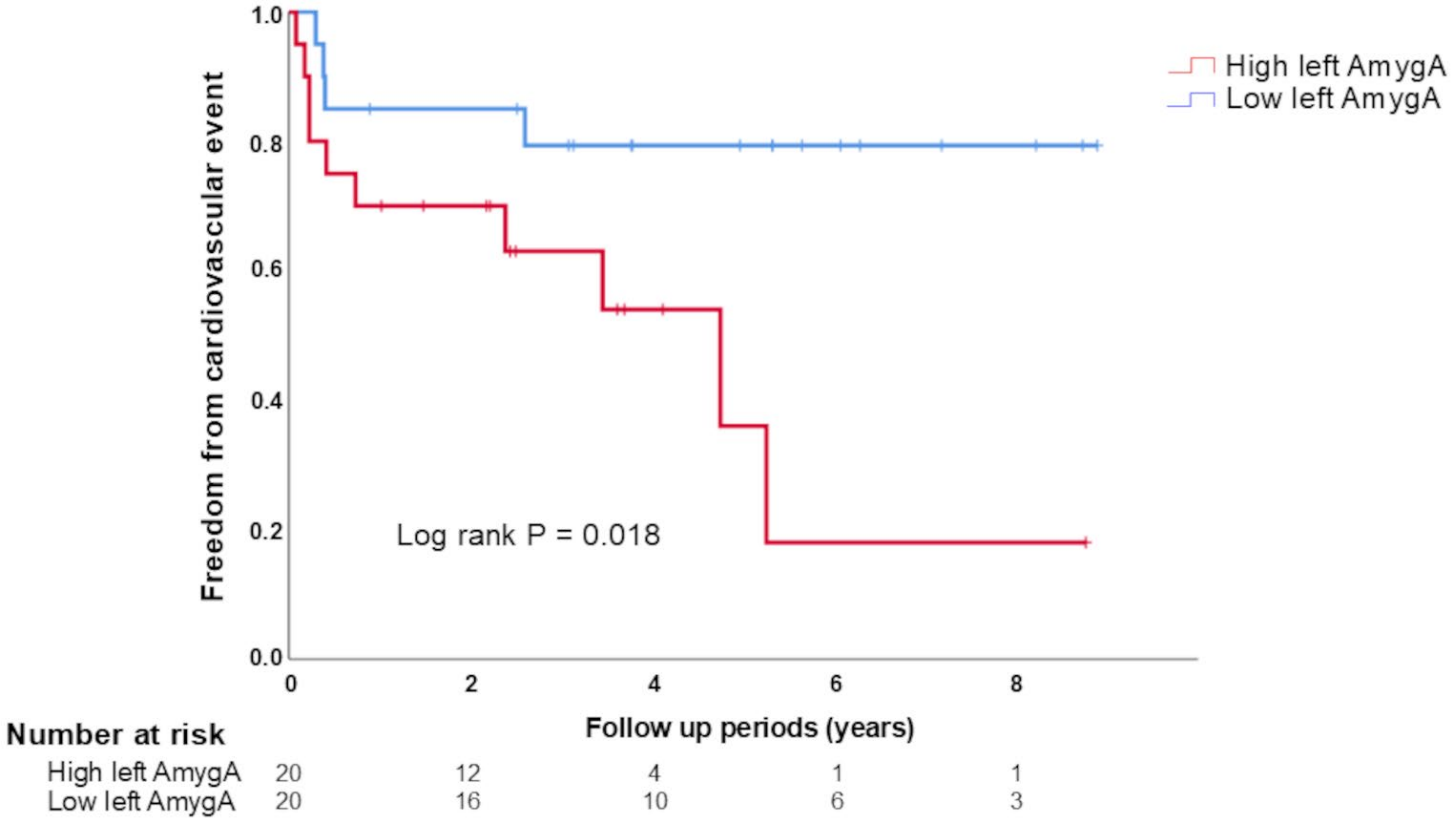
Kaplan–Meier analyses of the freedom from cardiovascular events (VT/VF and heart failure hospitalization) VT: ventricular tachycardia; VF: ventricular fibrillation

We divided all patients into two cohorts (high or low left-AmygA group), considering that the value of the left-AmygA was higher or lower than the median value estimated overall (Table 3). Body weight and body mass index (BMI) were lower, and the use of ACEi/ARB was less prevalent in the high-AmygA group than in the low-AmygA group. ECG results at baseline revealed that the QRS duration was significantly longer in the high left-AmygA group than in the low-left-AmygA group. During the follow-up period, 14 patients suffered from cardiovascular events (high left-AmygA group: 10 (50%) versus low left-AmygA group: 4 (20%)). Kaplan–Meier analysis revealed that patients in the high left-AmygA group demonstrated a high risk of cardiovascular events (log-rank *P* = 0.018) (Fig. 1). Table 4 presents the results of the univariate and multivariate analyses. Multivariate analyses revealed the left-AmygA augmentation (hazard ratio [HR] per 0.1 increase = 1.76, 95% CI = 1.12 − 2.87, *P* = 0.016) as the only independent predictor of cardiovascular events. Similarly, among 32 patients who underwent multiple PET scans, multivariate analyses revealed the mean left-AmygA on multiple PET scans as the only independent predictor of cardiovascular events (*P* = 0.011) (Table 5). The left-AmygA tended to be a VT/VF predictor when a similar analysis was conducted for VT/VF (*P* = 0.081).

**Table 3:** Comparison of patient characteristics between high and low left amygdala activity groups

	High left AmygA (> 1.097) *n* = 20	Low left AmygA (< 1.097) *n* = 20	*P* value
Age, years	63.0 (53.4–68.3)	64.5 (59.3–69.0)	0.55
Male, *N* (%)	4 (20%)	7 (35%)	0.48
Height (cm)	159.1 ± 8.4	158.7 ± 9.0	0.89
**Weight (kg)**	**55.1 ± 8.0**	**61.8 ± 8.9**	**0.019**
**Body mass index**, **kg/m2**	**21.1 (20.1–22)**	**23.5 (21.6–26.6)**	**0.011**
Systolic blood pressure, mmHg	103 (91.5-120.8)	114 (91–123)	0.70
Heart rate, bpm	67.4 ± 10.7	67.5 ± 9.6	0.97
Therapy			
Maintenance dose of corticosteroids, mg/d	5.0 (5.0–5.0)	5.0 (5.0-6.9)	0.66
B-blocker, *N* (%)	8 (40%)	12 (60%)	0.34
**ACE inhibitor/ARB**, *N* (%)	**5 (25%)**	**14 (70%)**	**0.010**
Antiarrhythmic drugs class III, *N* (%)	0 (0%)	3 (15%)	0.23
PM, *N* (%)	4 (20%)	3 (15%)	1.0
ICD, *N* (%)	6 (30%)	5 (25%)	1.0
CRT-D, *N* (%)	6 (30%)	4 (20%)	0.72
Comorbidity			
Hypertension, *N* (%)	5 (25%)	11 (55%)	0.11
Chronic Kidney Disease, *N* (%)	3 (15%)	7 (35%)	0.27
Diabetes mellitus, *N* (%)	1 (5%)	6 (30%)	0.091
Dyslipidemia, *N* (%)	7 (35%)	7 (35%)	1.0
Atrial fibrillation, *N* (%)	2 (10%)	4 (20%)	0.66
NSVT, *N* (%)	4 (20%)	2 (10%)	0.66
Complete AVB, *N* (%)	10 (50%)	9 (45%)	1.0
History of VT/Vf, *N* (%)	4 (20%)	4 (20%)	1.0
Heart failure hospitalization, *N* (%)	3 (15%)	3 (15%)	1.0
CS diagnosis			0.66
Histological, *N* (%)	2 (10%)	4 (20%)	
Clinical, *N* (%)	18 (90%)	16 (80%)	
Abnormal uptake of ^18^F-FDG PET in the heart, *N* (%)	1 (5%)	3 (15%)	0.60
LV ejection fraction, (%)	45.3 ± 17.2	46.6 ± 13.1	0.79
ECG findings at baseline			
Corrected QT interval, ms	474.8 ± 47.2	463.4 ± 29.9	0.39
QRS duration, ms	**151.3 ± 29.1**	**131.4 ± 24.7**	**0.032**

Mean ± S.D

Abbreviations are shown in Table 1

**Table 4:** Univariate and multivariate analysis for prediction of cardiovascular events (in all 40 patients)

	cardiac events			
Analysis method	**Univariate**		**Multivariate**	
Parameters	OR (95% CI)	*p* value	OR (95% CI)	*p* value
Age	0.99 (0.96–1.05)	0.98	−	−
Male	0.82 (0.25–2.66)	0.74	−	−
Weight	0.95 (0.89–1.01)	0.079	−	−
Body mass index (/1kg/m2)	0.82 (0.64–0.99)	0.077	−	−
Systolic blood pressure (/1mmHg)	**0.96 (0.91–0.99)**	**0.022**	0.96 (0.90–1.02)	0.26
Heart rate (/1 bpm)	0.96 (0.90–1.02)	0.22	−	−
ICD/CRT-D	**7.59 (1.69-34.0)**	**0.008**	2.28 (0.27-19.0)	0.45
LV ejection fraction (/1%)	0.98 (0.95–1.02)	0.33	−	−
QRS duration (/1ms)	1.01 (0.99–1.02)	0.48	−	−
Corrected QT interval (/1ms)	1.01 (0.99–1.03)	0.065	−	−
Beta-blockers	0.94 (0.33–2.69)	0.91	−	−
Antiarrhythmic drugs class III	2.99 (0.65–13.7)	0.16	−	−
ACE inhibitor/ARB	0.77 (0.27–2.21)	0.62	−	−
History of VT/Vf	**3.95 (1.37–11.5)**	**0.011**	2.76 (0.83–9.20)	0.099
History of heart failure hospitalization	0.97 (0.22–4.36)	0.97	−	−
NSVT	2.49 (0.67–9.25)	0.17	−	−
Complete AVB	0.86 (0.30–2.50)	0.79	−	−
Atrial fibrillation	0.84 (0.19–3.75)	0.81	−	−
Abnormal uptake of ^18^F-FDG PET in the heart	1.52 (0.34–6.82)	0.58	−	−
Amygdala activity				
Left (/0.1)	**1.75 (1.14–2.72)**	**0.010**	**1.76 (1.12–2.87)**	**0.016**
High left amygdala activity (> 1.097)	**3.82 (1.17–12.5)**	**0.027**	−	−
Right (/0.1)	**1.39 (0.98–1.90)**	**0.049**	−	−
High right amygdala activity (> 1.056)	3.18 (0.98–10.3)	0.053	−	−

Abbreviations are shown in Table 1

**Table 5:** Univariate and multivariate analysis for prediction of cardiovascular events (Patients who underwent multiple PET-CTs *N* = 32)

	cardiac events			
Analysis method	**Univariate**		**Multivariate**	
Parameters	OR (95% CI)	*p* value	OR (95% CI)	*p* value
Age	0.99 (0.96–1.06)	0.99	−	−
Male	1.24 (0.36–4.27)	0.74	−	−
Weight	0.93 (0.86-1.00)	0.069	−	−
Body mass index (/1kg/m2)	0.79 (0.57–0.99)	0.085	−	−
Systolic blood pressure (/1mmHg)	**0.95 (0.90–0.99)**	**0.028**	0.95 (0.87–1.02)	0.19
Heart rate (/1 bpm)	0.98 (0.90–1.05)	0.64	−	−
ICD/CRT-D	**11.5 (1.47-90.0)**	**0.020**	1.95 (0.15–26.1)	0.61
LV ejection fraction (/1%)	0.97 (0.93–1.01)	0.10	−	−
QRS duration (/1ms)	1.01 (0.99–1.03)	0.23	−	−
Corrected QT interval (/1ms)	1.02 (1.00-1.03)	**0.034**	1.01 (0.99–1.03)	0.30
Beta-blockers	1.56 (0.46–5.36)	0.48	−	−
Antiarrhythmic drugs class III	1.82 (0.23–14.4)	0.57	−	−
ACE inhibitor/ARB	0.80 (0.24–2.62)	0.71	−	−
History of VT/Vf	**3.45 (1.05–11.3)**	**0.042**	2.37 (0.61–9.30)	0.22
History of heart failure hospitalization	1.79 (0.38–8.43)	0.46	−	−
NSVT	2.14 (0.45–10.1)	0.34	−	−
Complete AVB	0.74 (0.22–2.54)	0.63	−	−
Atrial fibrillation	0.84 (0.18–3.93)	0.83	−	−
Abnormal uptake of ^18^F-FDG PET in the heart	2.70 (0.57–12.8)	0.21	−	−
Amygdala activity (mean value on multiple PET-CT scans)				
Left (/0.1)	**1.93 (1.07–3.59)**	**0.031**	**2.27 (1.24–4.47)**	**0.011**
High left amygdala activity (> 1.086)	3.39 (0.90–12.8)	0.072	−	−
Right (/0.1)	1.91 (0.95–3.80)	0.064	−	−
High right amygdala activity (> 1.127)	**5.63 (1.21–26.2)**	**0.028**	−	−

Abbreviations are shown in Table 1

## Discussion

### Main findings

The major results of the present study included the following: (1) high left-AmygA in patients with CS is related to cardiovascular events. (2) The AmygA value on the first PET scan was equivalent to the mean AmygA value on multiple PET scans in each patient.

### Association between left-AmygA and cardiovascular events

Chronic stress triggers a serial pathway that involves heightened stress-related neural network activity (SNA) (notably involving heightened AmygA), causing downstream sympathetic stimulation and leukopoiesis, atherogenesis, and atherosclerotic inflammation, culminating in major adverse cardiovascular events (MACE) [10, 16]. The previous study indicated that the benefit of light/moderate (vs. none/minimal) alcohol consumption on cardiovascular disease risk in part stems from its ability to attenuate SNA [9]. In contrast, Mikail et al. revealed that SNA imaging was a robust and independent predictor of all-cause mortality, but its prognostic value for MACE was less evident [11]. Until now, the relationship between cardiovascular events and SNA as calculated by AmygA has not been fully clarified. This is partly because previous studies included patients without cardiac disease, and the influence of imaging timing on SNA measurement was unclear.

The present study indicated that high left-AmygA was associated with a higher incidence of cardiovascular events, primarily VT/VF, in patients with CS. Mikail et al. revealed that compared with mean and right AmygAs, left-AmygA demonstrated a stronger association with MACE or overall mortality. Further, previous reports indicating a more prevalent role of the left amygdala in emotion processing robustly supported revealed this apparent laterality in SNA [11].

Although the present study suggests the usefulness of SNA measurement in patients with CS, further investigation is required to determine its use in other cardiac diseases. A previous study enrolled patients with ischemic heart failure and revealed that hypometabolism in several brain regions, including amygdala, was related to fatal arrhythmias [17]. They indicated that the injured myocardium causes functional disorders in the central autonomic network via afferent fibers in heart failure, and the hypometabolism in the autonomic nervous system-related brain regions induced major arrhythmic events through sympathetic-parasympathetic imbalance. Therefore, the effect of AmygA on ventricular arrhythmias may vary based on the baseline cardiac condition, such as underlying heart disease.

The present study identified a strong correlation between the left amygdala and cardiovascular events, which is consistent with previous reports [16, 18]. AmygA has been relatively stable over time [19] and is associated with an individual’s perceived stress [10]. Most previous studies have depended on a single measurement of SNA for their results, and 80% of all patients in this study underwent multiple PET examinations. This study demonstrated that AmygA showed little change during the disease course. Moreover, the association between cardiovascular events and AmygA was similar whether we utilized the average of values from multiple imaging or only the results of the first PET scan. These results suggest that, in patients with CS, AmygA measured by FDG-PET would be a useful prognostic biomarker regardless of the timing of imaging.

Notably, patients with higher AmygA had lighter weight and BMI and longer QRS duration, in addition to using ACEi/ARB less frequently. Recent data indicate that angiotensin receptors are expressed within the brain as well as the periphery and are found in brain regions involved in threat processing and fear conditioning, such as the amygdala, hippocampus, and prefrontal cortex [20]. Losartan administration has been reported to suppress amygdala activation and consequently improve anxiety responses [21]. ACEi/ARB have been reported to reduce the risk of sudden cardiac death in patients with heart failure with reduced ejection fraction [22, 23]. Therefore, AmygA reduction by ACEi/ARB may be involved in ventricular tachycardia suppression. Sympathetic activity can also influence the body weight. Increase in sympathetic activity causes reduction of food intake and increase of energy expenditure, thereby reducing body weight [24]. Thus, amygdala overactivation may cause lighter body weight in these patients via sympathetic hyperactivity. These factors may have affected the results, but they were not determined as risk factors for cardiovascular events in this study. The sample size is too small; thus, further studies are warranted to verify the association between these factors and AmygA.

A previous study revealed myocardial inflammation on FDG-PET images as a potent predictor of ventricular arrhythmias [25]. Herein, only four (10%) patients confirmed myocardial inflammation on the first PET, and we found no significant association between myocardial inflammation and cardiac events. The analyzed PET images were limited to after prednisolone induction; thus, low-frequency myocardial inflammation and lacking association between myocardial inflammation and cardiac events among our patients are partially because of therapeutic effects. Determining if AmygA would provide independent prognostic information in addition to that offered by the cardiac PET analysis in de novo patients warrants further investigation.

### Limitations

This study has several limitations. First, this was a retrospective single-center study with a small sample size. The number of cardiac events (*n* = 14) is extremely low for a multivariate analysis, which may have affected the results. Second, this study included only patients with CS; thus, the association between high left AmygA levels and cardiovascular events remains unclear in patients with other cardiac diseases. Furthermore, we cannot conclude that the left-AmygA in patients with CS who had higher cardiac events is higher than that in healthy controls, because we didn’t perform FDG-PET in control. Third, this study included 12 (30%) patients without CIEDs, in whom VT/VF events may have been missed. In 28 (70%) ICD/CRT-D/PM patients, differences in the setting of the minimum detection rate may have affected the results. However, univariate analysis revealed that the high left-AmygA was the only independent risk factor for cardiovascular events, regardless of including only 21 patients with ICD/CRT-D or 28 patients with ICD/CRT-D/PM **(Supplemental Table 1)**. Fourth, the Cox analysis and Kaplan-Meier method in this study use the date of cardiac sarcoidosis diagnosis as the start date for follow-up. Since seven patients experienced cardiac events before undergoing imaging, the PET imaging date could not be used as the start date to in the Cox analysis or Kaplan-Meier method. Consequently, the possibility cannot be ruled out that the deterioration of cardiac disease or therapeutic interventions after the diagnosis date influenced the amygdala activity value. However, even when limiting the analysis to the remaining 33 patients and using the PET imaging date as the start date, a trend remained in which left-AmygA was a predictor of cardiovascular events (*P* = 0.058). Fifth, socioeconomic status, such as low income and high crime, and lifestyle factors, including alcohol consumption and exercise, were not investigated in this study. These may have affected the results because they may modulate SNA [26, 27, 28]. Further prospective studies that involve a larger number of patients monitored with ICD/CRT-D, including those with other cardiac diseases, are warranted to confirm the results of this study. A causal association between cardiovascular events and AmygA could be clarified in the future with the addition of sympathetic hyperactivity evaluation (e.g., nocturnal heart rate and heart rate viability) using CIED.

## Conclusion

The left-AmygA value by PET remains relatively stable during the follow-up period in patients with CS, and higher activity may be related to cardiovascular events.

## Electronic supplementary material

Below is the link to the electronic supplementary material.

Supplemental Figure 1: Comparison of AmygA values on the first PET with those on multiple PETs in 32 patients who underwent multiple PET examinations. No significant differences were found in the right side, left side, or bilateral means. Box plots show the median, interquartile range, and range of the results. The circles represent measurements outside the 1.5 × interquartile range. (AmygA, amygdala activity; PET: positron emission tomography)

Supplemental Figure 2: Distribution of AmygA values on first PET in all patients. (AmygA, amygdala activity; PET: positron emission tomography)

Supplemental Table 1: Univariate and multivariate analysis for prediction of cardiovascular events (ICD/CRT-D patients *N*=21)

## Data Availability

The datasets generated and/or analyzed during the current this study are available from the corresponding author upon reasonable request.

## Abbreviations

ACE inhibitor/ARB: Angiotensin Coverting Enzyme Inhibitor/angiotensin receptor blocker
AmygA: Amygdala activity
AVB: Atrioventricular block
BMI: Body mass index
CRT: Cardiac resynchronized therapy
CIED: Cardiovascular implantable electronic device
CS: Cardiac sarcoidosis
ECG: Electrocardiography
ICD: Implantable Cardioverter Defibrillator
LV: Left ventricular
MACE: Major adverse cardiovascular events
NSVT: Nonsustained ventricular tachycardia
PM: Pacemaker
SNA: Stress-related neural network activity
VT: Ventricular tachycardia
VF: Ventricular fibrillation
