# Supplementary figures and images for: Relation between resting amygdala activity and cardiovascular events in patients with cardiac sarcoidosis
Source: Eur J Nucl Med Mol Imaging. 2025 Apr 14;52(11):4224–32. doi: 10.1007/s00259-025-07266-3 (PMC12396973; doi:10.1007/s00259-025-07266-3)

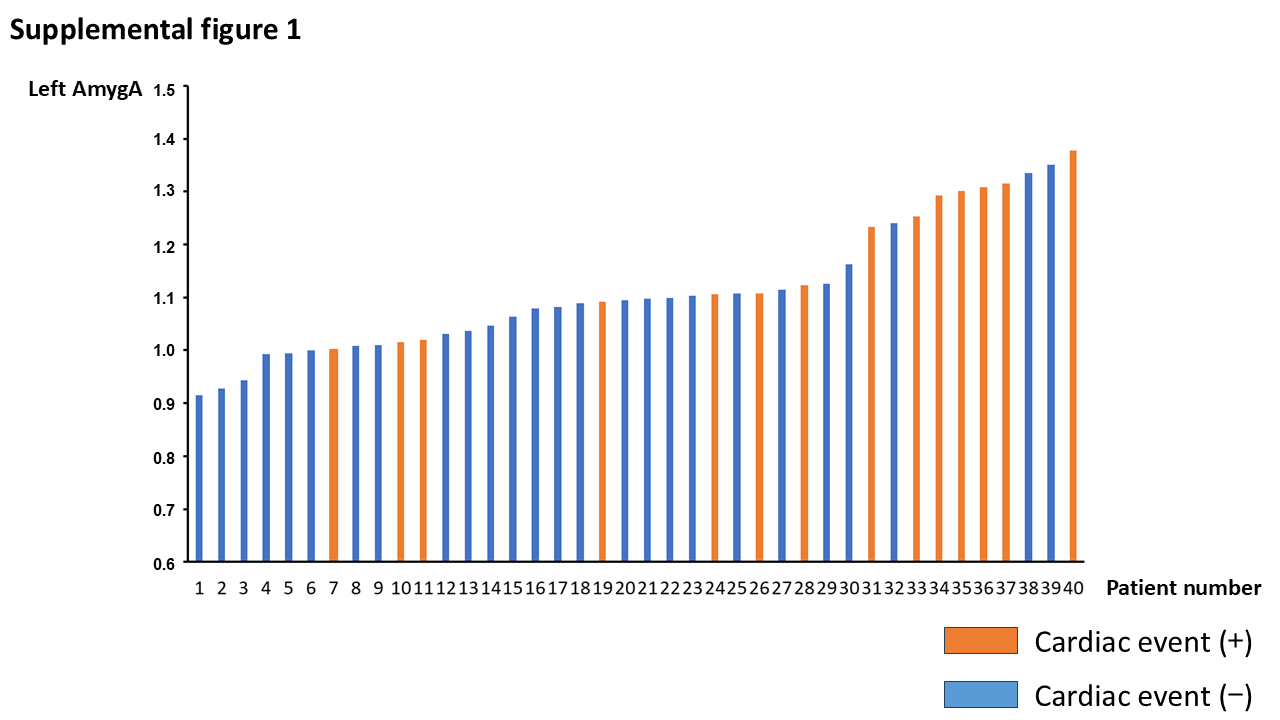

Supplement: Supplementary file 1 — Supplemental Figure 1: Comparison of AmygA values on the first PET with those on multiple PETs in 32 patients who underwent multiple PET examinations. No significant differences were found in the right side, left side, or bilateral means. Box plots show the median, interquartile range, and range of the results. The circles represent measurements outside the 1.5 × interquartile range. (AmygA, amygdala activity; PET: positron emission tomography) [file 259_2025_7266_MOESM1_ESM.png]

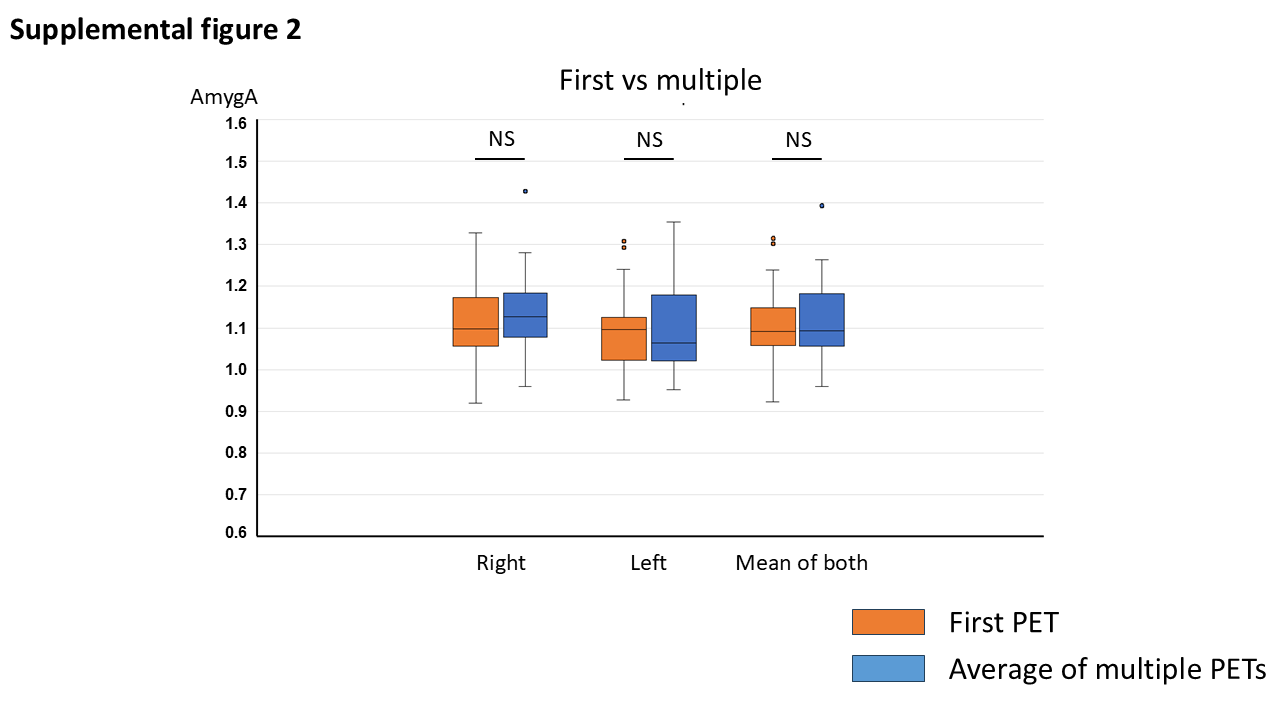

Supplement: Supplementary file 2 — Supplemental Figure 2: Distribution of AmygA values on first PET in all patients. (AmygA, amygdala activity; PET: positron emission tomography) [file 259_2025_7266_MOESM2_ESM.png]
