# Supplementary material for: Relation between resting amygdala activity and cardiovascular events in patients with cardiac sarcoidosis
Source: Eur J Nucl Med Mol Imaging. 2025 Apr 14;52(11):4224–32. doi: 10.1007/s00259-025-07266-3 (PMC12396973; doi:10.1007/s00259-025-07266-3)
Supplement: Supplementary file 3 — Supplemental Table 1: Univariate and multivariate analysis for prediction of cardiovascular events (ICD/CRT-D patients N=21) [file 259_2025_7266_MOESM3_ESM.docx]

**Supplemental table 1. Univariate and multivariate analysis for prediction of cardiovascular events (ICD/CRT-D patients N=21)**

|  | **cardiac events** | |
| --- | --- | --- |
| Analysis method | **Univariate** | |
| Parameters | OR (95% CI) | *p* value |
| Age | 0.94 (0.85-1.02) | 0.15 |
| Male | 1.52 (0.41-5.64) | 0.53 |
| Weight | 0.98 (0.91-1.06) | 0.67 |
| BMI (/1kg/m2) | 0.88 (0.63-1.12) | 0.39 |
| Systolic blood pressure, mmHg | 0.97 (0.90-1.02) | 0.26 |
| Heart rate, bpm | 0.97 (0.91-1.04) | 0.39 |
| LV ejection fraction (/1%) | 1.01 (0.97-1.05) | 0.61 |
| QRS duration (/1ms) | 0.99 (0.97-1.01) | 0.54 |
| Corrected QT interval (/1ms) | 1.00 (0.98-1.02) | 0.99 |
| Beta-blockers | 0.82 (0.26-2.54) | 0.72 |
| Antiarrhythmic drugs class III | 4.66 (0.85-25.7) | 0.077 |
| ACE inhibitor/ARB | 0.93 (0.29-2.93) | 0.90 |
| History of VT/Vf | 1.76 (0.55-5.60) | 0.34 |
| History of heart failure hospitalization | 0.60 (0.13-2.80) | 0.52 |
| NSVT | 2.85 (0.70-11.5) | 0.14 |
| Complete AVB | 0.71 (0.21-2.36) | 0.57 |
| Atrial fibrillation | 0.39 (0.05-3.07) | 0.37 |
| Abnormal uptake of ^18^F-FDG PET in the heart | 2.49 (0.51-12.1) | 0.26 |
| Amygdala activity |  |  |
| Left (/0.1) | 1.38 (0.86-2.21) | 0.17 |
| **High left amygdala activity (>1.104)** | **4.07 (1.16-14.2)** | **0.028** |
| Right (/0.1) | 1.10 (0.67-1.67) | 0.69 |
| High right amygdala activity (>1.129) | 1.23 (0.39-3.83) | 1.23 |

Abbreviations are shown in Table 1.
